# Gastric pericardial fistula in a bariatric patient

**DOI:** 10.1016/j.ijscr.2024.110512

**Published:** 2024-10-23

**Authors:** Alison J. Lehane, Olivia Fukui, Adolfo “Fuzz” Fernandez

**Affiliations:** Department of General Surgery, Atrium Health Wake Forest Baptist, Winston-Salem, NC, United States of America

**Keywords:** Gastric pericardial fistula, Roux-en-y, Bypass, Complications, Marginal ulcer

## Abstract

**Introduction and importance:**

Bariatric surgery has positively affected the lives many by improving their overall health with weight loss. Unfortunately, there are complications to bariatric surgery including anastomotic leaks, bowel obstructions, hernias, and ulcers. We present a case report of a gastric-pericardial fistula in the setting of a marginal ulcer of a patient with prior Roux-en-Y gastric bypass (RNYGB).

**Case presentation:**

The patient is a 71 year old female with a history of diabetes, hypertension, hypothyroidism and arthritis requiring many prior joint replacements and leaving the patient partially disabled requiring assistances daily. In addition, she had a RNYGB5 years prior to presentation. She presented to an outside hospital with shortness of breath, cough, and chest pain for 3 days prior. A CTA revealed pneumopericardium and small pericardial effusion.

**Clinical discussion:**

She was transferred to us for VATS & EGD with CT surgery which revealed a pericardial gastric fistula. Bariatric surgery team was then consulted and discovered risk factors including smoking and NSAID use. Esophageal stent was placed by gastroenterology and as her course was complicated by COVID pneumonia requiring ICU transfer and intubation. Once recovered, surgical intervention was planned with bariatric surgery involving laparoscopic fistula takedown, gastrojejunostomy revision and G tube placement.

**Conclusion:**

Gastric-pericardial fistula is a rare complication of bariatric surgery with a high mortality rate amongst the few cases reported. Early detection and multidisciplinary teamwork is necessary for good patient outcomes. It is important to emphasize risk factors for ulcers to patients and the serious complications that can occur.

## Highlights

• Bariatric surgery has improved many lives, but complications can include anastomotic leaks, bowel obstructions, hernias, and ulcers.
• Gastric-pericardial fistula is a rare, high-risk complication of bariatric surgery with significant mortality.
• Early detection and a multidisciplinary approach are crucial for improving patient outcomes.

## 1 Introduction

Bariatric surgery has revolutionized the management of obesity and its associated comorbidities, offering significant and sustained weight loss along with improvements in metabolic diseases and overall quality of life [1,2]. However, despite its benefits, bariatric surgery is not devoid of complications, some of which can be severe and life-threatening [3,4].

One such rare yet potentially catastrophic complication is the development of gastric pericardial fistula. This condition involves an abnormal communication between the gastric or esophageal lumen and the pericardium, leading to the leakage of gastric contents into the pericardial space [5,6]. While the exact pathophysiology of gastric pericardial fistula formation remains poorly understood, it is believed to be multifactorial, often occurring secondary to chronic irritation of the gastric mucosa, ulcer formation, and subsequent erosion into adjacent structures [6].

The clinical presentation of gastric pericardial fistula can vary widely, ranging from nonspecific symptoms such as chest pain, shortness of breath, and cough to more severe manifestations such as pericardial effusion, cardiac tamponade, and sepsis [7,8]. Prompt recognition and diagnosis of this condition are paramount, as delayed intervention can result in significant morbidity and mortality. In this report, we present a case of gastric pericardial fistula in a 71-year-old female with a history of RNYGB, emphasizing the challenges encountered in its diagnosis and management. This work has been reported in line with SCARE Criteria [9].

## 2 Case presentation

A 71-year-old female with a history of diabetes, hypertension, hypothyroidism, and arthritis presented with symptoms of shortness of breath, cough, and chest pain. She had undergone RNYGB five years prior. She presented to an outside hospital with shortness of breath, cough, and chest pain for 3 days prior. A computed tomography angiography (CTA) revealed pneumopericardium and small pericardial effusion, thus she was transferred to our institution for further evaluation by the cardiothoracic surgery team.

**Fig. 1:**
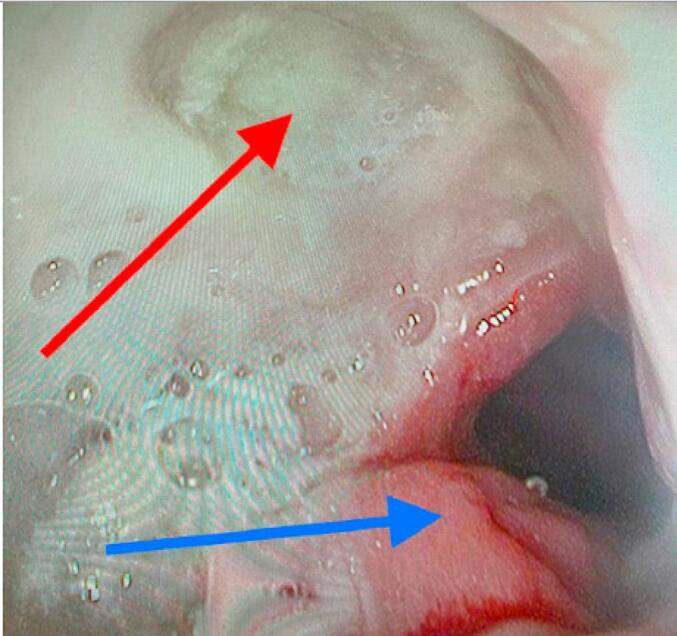
Endoscopic picture of gastric pericardial fistula. Red arrow: Gastric ulcer covered with exudates. The arrow head points to an area that moved back and forth with the heart beat suggesting communication with the pericardial space. Blue arrow: Gastric mucosa. (For interpretation of the references to colour in this figure legend, the reader is referred to the web version of this article.)

She was placed on broad spectrum antibiotic and underwent an esophagram as she reported recently undergoing diagnostic endoscopy, thus there was concern for possible esophageal injury contributing to the presentation, but this showed no abnormalities. Patient then underwent video assisted thoracoscopic surgery (VATS), pericardial window and esophagogastroduodenoscopy (EGD) with cardiothoracic surgery. The pericardium was incised and turbid fluid was collected. A pericardial window was created. EGD was then performed and while there was no evidence of ulcerations or perforations in the esophagus, there was a gastric ulcer with an apparent fistulous tract covered with exudates that moved back and forth with the heartbeat suggesting communication with the pericardium. (Fig. 1) A CT of the abdomen and pelvis was then performed postoperatively with oral contrast which showed enteric contrast opacifying the right pericardium indicating marginal ulceration and subsequent fistualization between the pericardium and the gastrojejunostomy anastomosis. There was noted to be decreased pericardial gas now that the mediastinal drain was in place.

Our bariatric surgery team was then consulted. Upon further discussion with the patient, she stated she smokes 4–5 cigarettes daily and reports taking NSAIDs for backpain frequently. At this time, she was placed on total parenteral nutrition (TPN) for early nutritional support. Gastroenterology (GI) was then consulted and placed an esophageal stent. The patient was improving but then unfortunately was found to be COVID positive and had worsening respiratory distress leading to intensive care unit (ICU) transfer and intubation. Her condition improved and once recovered from her COVID, she was taken to the operating room for laparoscopic gastrojejunostomy revision and gastrostomy tube placement.

Laparoscopically, the gastrojejunostomy was freed from the adhesions to the liver, pericardium and bypassed stomach using blunt and Harmonic dissection. There was a large perforation of the gastrojejunostomy anteriorly. This was dissected from the pericardium. GI performed an upper endoscopy and removed the covered stent from her pouch and Roux. The remaining Roux was dissected from the gastrojejunostomy using the Harmonic. The posterior adhesions between the pouch and bypassed stomach were lysed in order to expose the gastrotomy. The proximal portion of the Roux was transected using an Echelon white load stapler and it was removed as specimen. An end to side gastrojejunostomy was performed over a 36 French Bougie using 2–0 Vicryl suture. A bowel clamp was placed proximally on the Roux limb so an intraoperative gastroscopy could be performed. The visualized portions of the esophagus, pouch, anastomosis, and the Roux limb were intact without evidence of ulcer, bleeding or defects. Simultaneously, we performed an air leak test which was negative.

An 18 French foley was used as the gastrostomy tube; it was witzeled into the bypassed stomach. Omentum was tacked above the gastrojejunostomy using 2–0 Vicryl suture to separate it from the pericardium. A drain was placed above the anastomosis. On post operative day 8, the patient decompensated requiring ICU transfer. Further work up included a CT abdomen/pelvis which revealed a gastric outlet obstruction of her bypassed stomach causing massive dilation of the bypassed stomach likely due to spontaneous hyperinflation of the G-tube balloon. The balloon was deflated and the obstruction resolved. She was advanced to a full liquid diet and TPN was weaned. She was discharged to a rehabilitation facility and has been doing well since.

## 3 Discussion

Gastric pericardial fistula is a rare but serious complication of bariatric surgery, with only a handful of cases reported in the literature [8,10,11]. While the majority of gastric pericardial fistulas occur following Roux-en-Y gastric bypass, cases have also been reported following other surgical procedures such as Nissen fundoplication and esophagectomy [7,12].

The pathogenesis of gastric pericardial fistula formation is thought to involve a combination of mechanical, ischemic, and inflammatory factors. Chronic irritation of the gastric mucosa, often exacerbated by factors such as smoking and nonsteroidal anti-inflammatory drug (NSAID) use, can lead to the development of gastric ulcers [6]. In the setting of bariatric surgery, altered anatomy and impaired blood supply to the gastric remnant may further predispose patients to ulceration and subsequent fistula formation. Diagnosing gastric pericardial fistula can be challenging, as its clinical presentation can mimic other more common conditions such as myocardial infarction, pulmonary embolism, and pneumonia. Imaging modalities such as computed tomography (CT) scans, esophagograms, and upper endoscopy play a crucial role in establishing the diagnosis, with findings suggestive of fistulous tracts or pericardial effusion raising suspicion for this condition [5].

Management of gastric pericardial fistula typically involves a combination of medical therapy, endoscopic intervention, and surgical repair. In the acute setting, patients may require resuscitation with intravenous fluids, antibiotics, and hemodynamic support. Endoscopic interventions such as stent placement or fibrin glue injection may be attempted to temporarily seal the fistulous tract and stabilize the patient prior to definitive surgical repair [6,8,10]. Surgical intervention is often necessary to address the underlying fistulous tract and prevent further leakage of gastric contents into the pericardial space. This may involve procedures such as pericardial window creation, gastrojejunostomy revision, and placement of feeding tubes to divert gastric contents away from the affected area [6,7]. In our case, the patient underwent multiple procedures including VATS, endoscopic stenting, laparoscopic gastrojejunostomy revision, and gastrostomy-tube placement, highlighting the complexity of managing this condition.

## 4 Conclusion

Gastric pericardial fistula is a rare but serious complication of bariatric surgery that requires prompt diagnosis and intervention. Multidisciplinary collaboration involving cardiothoracic surgery, gastroenterology, and bariatric surgery is essential for optimal management. Despite challenges, favorable outcomes can be achieved with timely intervention and appropriate supportive care.

## Author contribution

Alison Lehane: study concept, writing of paper, data analysis

Olivia Fukui: reviewing and editing of paper, data collection

Adolfo Fernandez: reviewing and editing of paper, study concept.

## Consent

Written informed consent was obtained from the patient for publication and any accompanying images. A copy of the written consent is available for review by the Editor-in-Chief of this journal on request.

## Ethical approval

Ethics approval is not required for case reports or case series at my research institution at Atrium Health Wake Forest Baptist Medical Center.

## Funding

None.

## Guarantor

Adolfo Fernandez.

## Declaration of competing interest

The authors have no conflicts of interest.
